# Ictus Solaris—Rheumatism—Extensive Thoracic Disease

**Published:** 1826-07

**Authors:** 


17. *Ictus Solaris*. Mr. Brown, Surgeon of the 2d Dragoon Guards, has related an interesting case of this affection, which took place dnring the hot weather of August last, in the person of a farrier of the regiment—who, having been drinking freely, had run about in the sun, with his head uncovered, in the midst of which exercise he dropped down suddenly, as if he had been shot. Mr. Brown found him extended, senseless, and motionless—his breathing free and natural—skin hot—countenance flushed—vessels of the eye turgid—pupils dilated, and iris not obedient to the light—pulse very full and firm, but not rapid—jaws clenched—fæces and urine passing involuntarily. There was no time to be lost. Large detractions of blood were made from the arm, temporal artery, and jugular vein, aided by saline glysters, refrigerants, and cool air, with shower-bath every four hours. He lost, in the commencement, 112 ounces of blood—but it was five or six days before any thing more than a very slight amendment could be perceived. At that period, he began to shew signs of sensibility and consciousness of surrounding objects. On recovering a little farther, it was found that he had complete hemiplegia of the left side. In about six weeks he was able to join his regiment, and attend to his duty, the hemiplegia having almost entirely disappeared

In this case it is evident that apoplectic fulness of the vessels must have first obtained, and that some effusion—probably of very trifling extent, took place in the right side of the brain.

*Rheumatism*. The dragoons are more subject to this distressing disease than the foot soldiers. The greater number of cases, in Mr. Brown's practice, were without febrile excitement. External applications were of little avail. As an internal medicine, the colchicum, in increasing doses, had a decided superiority over others. "But where there was no apparent phlogistic diathesis present, I have lately found that mercurial vapour, applied to the whole surface night and morning, until a slight action is perceptible, has an unequivocal advantage over every other remedy I have yet tried—giving tone and activity to the circulation, requiring no muscular exertion in its use, being cleanly and safe in its application."

*Extensive Thoracic Disease*. Mr. Brown has given this case of the late quarter-master of the Bays, who appears to have been early afflicted with rheumatism, but, for the last 7 years with gout, which came on in regular autumnal attacks. In October, 1823, a fit was repelled by some nostrum, and was succeeded by pain in the præcordia and stomach, the lungs and heart participating. This retrocession required active bleeding, warm baths, blisters, opiates, and antispasmodics, for the space of six days. The prominent symptoms were subdued, but severe effects remained. In the autumn of 1824, he was noted in Mr. Brown's Journal, as affected with constant cough—copious expectoration—suffocating respiration—inability to lie down—full pulse, with intermissions—vertigo, palpitation—great dyspnœa on going up stairs—constant pain in the left breast, with a frequent painful constriction across the whole chest, extending around each deltoid muscle. The action of the heart was visibly laborious, extending over a space of six or seven inches in diameter; the whole arterial system vibrating in consonance with the central organ. In this state, more or less aggravated, he continued until May 1825, when he died suddenly, after shaving and dressing himself.

*Dissection.* The left side of the thorax was emphysematous, the muscles flabby and easily lacerable, and the ribs soft. Extensive adhesions obtained between the lungs and the pleura costalis—the lungs themselves were hepatised in some places, and generally studded with tubercles. The heart was enormously enlarged, flabby, and covered with fat—six ounces of serum in the cavity of the pericardium—the parietes of the right chambers were, in some places, so thin, as to be nearly transparent—the pericardium itself diseased, and various other morbid appearances in the chest. The coats of the stomach were thickened, and the internal surface of a chocolate colour.—*Med. and Phys. Journal, March,* 1826.

The above specimen of thoracic, and especially of cardiac disease, is now familiar to almost every attentive observer, as resulting from the *extension,* as well as *metastasis,* of rheumatism from external to internal parts.

